# Uremic sarcopenia: the role of intramuscular adipose tissue as a potential early identifier

**DOI:** 10.3389/fmed.2024.1372668

**Published:** 2024-11-01

**Authors:** Annalisa Noce, Maria Josè Ceravolo, Paola Gualtieri, Giulia Marrone, Lorenzo Romano, Amir Shoshi, Manuela Di Lauro, Antonino De Lorenzo

**Affiliations:** ^1^Department of Systems Medicine, University of Rome Tor Vergata, Rome, RM, Italy; ^2^UOSD Nephrology and Dialysis, Policlinico Tor Vergata, Rome, RM, Italy; ^3^Department of Clinical Sciences, Catholic University Our Lady of Good Counsel, Tirana, Albania; ^4^Section of Clinical Nutrition and Nutrigenomics, Department of Biomedicine and Prevention, University of Rome Tor Vergata, Rome, RM, Italy; ^5^Programma Clinico di Tipo A “Nutrizione Clinica”, Policlinico Tor Vergata, Rome, RM, Italy; ^6^Program in Specialization in Nephrology, Catholic University Our Lady of Good Counsel, Tirana, Albania; ^7^Nuova Clinica Annunziatella, Rome, RM, Italy

**Keywords:** uremic sarcopenia, dialysis patients, intramuscular adipose tissue, parathyroid hormone, sarcopenia index, body composition analysis

## Abstract

**Introduction:**

Sarcopenia is a chronic pathological condition, first defined in 2010 and revised in 2018. The most recent definition of sarcopenia focuses mostly on “low muscle strength.” A secondary form of sarcopenia is represented by uremic sarcopenia (US), a condition that characterizes end-stage kidney disease (ESKD) patients. The intramuscular adipose tissue (IMAT) seems to impact negatively on muscle strength, as it would seem to replace muscle fibers with a non-contractile component. The study aims to compare body composition parameters—both standardized and innovative—related to the diagnosis of US in hemodialysis (HD) patients, stratified by sarcopenia diagnosis. Furthermore, the different indices of sarcopenia are compared in order to evaluate their predictive capacity.

**Methods:**

We analyzed 48 ESKD patients according to the sarcopenia diagnosis, obtained using dual-energy X-ray absorptiometry (DXA). Moreover, we assessed the presence of IMAT and calculated the sarcopenia index (SI).

**Results:**

For the study, the enrolled population was divided according to the sarcopenia diagnosis: no sarcopenic patients had higher transferrin (*p* = 0.03), total proteins (*p* = 0.04), and azotemia pre-dialysis (*p* = 0.05) values. On the contrary, atherogenic indices were lower in no sarcopenic patients. Moreover, we observed an indirect correlation between the SI and parathyroid hormone (PTH) (*p* = 0.00138, *R*^2^ = 0.54). Finally, we calculated the prevalence of sarcopenia and sarcopenia adjusted for IMAT. We showed a different prevalence between sarcopenia diagnosed with a standard index and an index adjusted for IMAT (*p* = 0.043). In conclusion, we believe that the most important result obtained is the indirect correlation between SI and PTH. These data corroborate the theories, in which PTH seems to play a central role in the cachexia genesis. Moreover, the SI adjusted for IMAT seems to be a more reliable parameter for the early identification of subjects at risk of developing US, allowing timely implementation of targeted therapeutic strategies.

## 1 Introduction

The first definition of “sarcopenia” was established in 2010 by the European Working Group on Sarcopenia in Older People (EWGSOP) (1), aiming to identify and manage individuals at risk of developing this pathological condition. In 2018, the EWGSOP updated this definition (EWGSOP2) according to new scientific and clinical evidence (2). The updated definition of sarcopenia emphasizes “low muscle strength” and in addition to this parameter, the assessment of muscle low quantity and quality is necessary for confirming the condition. A third parameter for determining the degree of sarcopenia is physical performance; poor performance indicates severe sarcopenia.

Sarcopenia is a physiological phenomenon that occurs throughout the lifespan and is characterized by adverse muscle changes (muscle failure) due to aging (3). Regarding public health costs, sarcopenia increases the risk of hospitalization and healthcare expenses (4). In fact, sarcopenia impacts significantly on the risk of falls and fractures, and it is related to cardiovascular (CV) and respiratory diseases. Moreover, it affects the ability to perform daily activities, leads to mobility disorders, and can contribute to cognitive impairments, thus reducing the quality of life and increasing the risk of death (5–9). In fact, it has been demonstrated by several studies that the reduction of muscle mass and fat-free mass (FFM) is associated with a more rapid cognitive decline in elderly subjects (10, 11).

A secondary form of sarcopenia is represented by the uremic sarcopenia (US). The “uremic milieu” is a typical condition of those patients affected by end-stage kidney disease (ESKD) (12). This condition leads to reduced muscle strength and an enhancement of morbidity and mortality for all causes. US is due to a “set” of progressive and cumulative effects induced by chronic kidney disease (CKD) on skeletal muscles. The US etiology is multifactorial, and it is linked to several factors, such as hormonal imbalances, vitamin D deficiency, metabolic acidosis, reduced protein intake, physical inactivity, hemodialysis (HD) treatment, accumulation of uremic toxins, secondary anemia, and chronic inflammatory state (13–19).

The overall prevalence of sarcopenia in ESKD patients is very high, reaching 50% in those undergoing HD (20). This muscle alteration, also in CKD patients, increases the risk of fractures and CV events, reduces overall survival, and negatively impacts on personal autonomy and quality of life (21, 22). The poor quality of life causes cognitive impairments, namely a potential alteration of the mood with consequent social isolation and deterioration of nutritional status. Depression, in association with the chronic inflammatory state, contributes to spontaneously reduce protein-energy intake, causing or worsening malnutrition up to, in the most severe cases, cachexia and anasarca status (23, 24). Over the years, different methods have been proposed for assessing body muscle mass. These include (i) anthropometric measurements, (ii) skinfold thickness measurements, (iii) bioimpedance analysis, (v) dual-energy X-ray absorptiometry (DXA) scan, and (vi) computed tomography (CT) scan (25). Anthropometric measurements of muscle mass, particularly mid-arm circumference taken at the midpoint of the upper arm, are among the most commonly used methods (26). These measurements include body weight, height, and circumferences (most notably waist and hip), providing a simple, fast, and cost-effective means of estimating body composition. Starting from the ratio between body weight (expressed in kg) and height squared (expressed in meters), it is possible to calculate the body mass index (BMI) (27). Although the BMI is constantly used for the assessment of body composition, it presents a limitation of not being able to differentiate between lean mass and fat mass (FM) (28). In fact, it should be considered a “crude” measurement of body size as it cannot distinguish between skeletal muscle and adipose tissue (29). Another tool to detect body composition is the thickness of skinfolds. This technique permits the estimation of the subcutaneous adipose tissue, detected in standardized points of the body such as the triceps, iliac crest, biceps, and calf (30–32). Given the relationship between subcutaneous fat and total body fat, it is considered that the result of the measurement of the folds is a good indicator of total body fat (33). The thickness of the adipose panniculus varies according to age, gender, and ethnicity.

In the late 1990s, Baumgartner et al. (34) suggested the use of a new parameter to determine sarcopenia. In fact, these authors stated that through a DXA examination, it is possible to obtain the bone-free mass (BFM) and FFM of the arms and legs, namely the appendicular skeletal muscle mass (ASM). Sarcopenia is defined when reduced values of ASM divided by height squared (ASM/height^2^) are observed, compared to the reference population matched by age and gender (34). Although the reduction of this index may indicate an increase in the risk of disability up to 3–4 times in elderly women and men (34), it may not always be reliable.

In human beings, different anatomical adipose depots have been described, performing several functions throughout their lifespan (35). The five different fat deposits in the human body are: subcutaneous, visceral, intermuscular, intramuscular, and bone adipose depots (36). Two of these classes of adipocytes, specifically the intermuscular and intramuscular ones, are present inside the skeletal muscle mass. The former are fat deposits present in the spaces between the skeletal muscles, while the latter comprises all the adipocytes that interpose between the various muscle fibers of the skeletal muscle (37). The early differentiation of these two adipose tissues occurs during the third trimester *in utero* and becomes evident in the postnatal period (38). The differentiation ends during late adolescence, when the intermuscular adipose tissue decreases and, on the contrary, the intramuscular adipose tissue (IMAT) increases (39), and the term “intramuscular fat” refers to the sum of the intermuscular fat and the IMAT (40). Several studies demonstrated that elevated levels of the IMAT are associated with decreased levels of strength and physical performance (41). In fact, the IMAT would seem to replace muscle fibers with a non-contractile component, and, in turn, the strength decreases. Other studies speculated that the IMAT could impair the contraction in the myofibers, caused by altered neuromuscular activation and muscle blood flow. In addition, it could cause the local release of pro-inflammatory adipokines (42–44). For this reason, it is important to consider not only the ASM but also the IMAT for a better assessment of the body composition. This index should be corrected for the IMAT to have a more predictive value.

This study aims to compare body composition parameters (both standard and innovative ones) in HD patients, stratified according to the diagnosis of sarcopenia. These parameters were assessed using bioelectrical impedance analysis (BIA), dual-energy X-ray absorptiometry (DXA), handgrip strength (HGS), and anthropometric measurements.

Furthermore, the different indices of sarcopenia were compared to evaluate their predictive capacity, both adjusted or unadjusted, for IMAT.

## 2 Patients and methods

### 2.1 Study population

The study was conducted on 48 patients (34 men and 14 women) undergoing chronic maintenance HD for at least 3 months, and it employed a cross-sectional design. The inclusion criteria were ESKD, age over 18 years, and being treated with the same dialysis technique for at least 3 months. Exclusion criteria were the presence of cancer, HIV^+^, HBsAg^+^, HCV^+^, and active autoimmune diseases. The patients followed three-times-weekly scheme with an average duration of 3.5 h both for convective and diffusive techniques.

The patients were enrolled at the Dialysis Unit of Policlinico Tor Vergata (Rome, Italy) and the Dialysis Center of Nuova Clinica Annunziatella (Rome, Italy).

The study protocol complied with the Declaration of Helsinki, and it was approved by the Ethics Committee of Fondazione Policlinico Tor Vergata (PTV) of Rome (experimentation register number 60/16).

Written informed consent for the study participation was obtained for all enrolled patients during the period September 2016- September 2017. At the time of enrolment, all selected patients underwent routine bloodwork and body composition assessment, using BIA, DXA, and anthropometric measurements. The HGS test was assessed to measure muscle strength.

### 2.2 Laboratory parameters

Blood sampling was performed at the long interdialytic interval. Measurement of laboratory parameters was performed using an automated hematology analyzer XE-2100 (Sysmex, Kobe, Japan) for the determination of hemoglobin (Hb). All routine parameters were determined using the Dimension Vista 1500 (Siemens Healthcare Diagnostics, Milano, Italy).

All laboratory parameters were analyzed according to standard procedures in the Clinical Chemical Laboratories of Fondazione PTV of Rome.

Parathyroid hormone (PTH) was analyzed using the Alinity i Intact PTH Reagent Kit (Alinity i, Abbott GmbH & Co. KG, Germany).

For evaluation of inflammatory status, we examined the C-reactive protein (CRP), the erythrocyte sedimentation rate (ESR), platelet-to-lymphocyte ratio (PLR), neutrophil-to-lymphocyte ratio (NLR), and lymphocyte-to-monocyte ratio (LMR) (45).

### 2.3 Body composition assessment

For all patients, the body composition assessment was performed. In particular, patients remained supine during the entire dialysis treatment and for an additional 15 min after the end of the HD session. A BIA was performed at the end of this period. The BIA electrodes were placed on the side of the patient where there was no vascular access for HD.

The BIA (QUANTUM V, RJL Systems, Bari Italy) was used to measure resistance (Rz), reactance (Xc), total body water (TBW), and extracellular body water (ECW) (46, 47).

Weight and height were assessed using a scale and stadiometer (Invernizzi, Rome, Italy), and parameters were recorded to the nearest 0.1 kg and 0.1 cm, respectively. The waist and hip circumferences were collected with a flexible and non-stretchable metric tape, and the waist-to-hip ratio was calculated.

The BMI was calculated as body weight (kg)/height (m)^2^ and classified according to the World Health Organization (WHO) guidelines (48).

The whole and segmental fat mass (FM), lean mass (LM), and bone mass (BM) were measured using a DXA (Primus, X-ray densitometer; software version 1.2.2, Osteosys Co., Ltd., Guro-gu, Seoul, Korea) (49). The intra- and inter-subject coefficient of variation (CV% = 100 standard deviations-SD/mean) was below 5% to validate a measure. The coefficients on this instrument for five participants scanned six times over 9 months were 2.2% for FM and 1.1% for FM and LM.

The DXA is a technique initially used for the determination of bone mineral density and subsequently used in soft tissue analysis for the evaluation of fat and lean mass (50). DXA is one of the most accurate methods for measuring total and district body FM and FFM (51). Currently, it is considered a technique that provides greater accuracy for the evaluation of LM and FM, expressed both in percentage value and in kilogram in the different body districts, allowing to precisely determine the areas of fat accumulation and the possible increase of LM in certain body districts (52).

Percentage FM was computed as whole FM in kilogram divided by the total mass of all tissues, including whole LM and BM, as follows: FM% = [whole FM/(whole FM + LM + BM)] × 100.

The IMAT was obtained according to the formulas provided by Bauer et al. (53, 54):

- Log (IMAT) = −2.21 + (0.12 × fat) + (−0.0013 × fat^2^) for women
- Log (IMAT) = −2.05 + (0.12 × fat) + (−0.0013 × fat^2^) for men.

BIA is a technique that analyzes R and Xc using a low-voltage electric current at a high frequency (50 kHz). The flow of the current allows us to estimate the body composition according to the water content in the human body (55, 56). The biological tissues of our body can act both as conductors and as insulators: all fluids and LM are excellent conductors, while the fat is a bad conductor and offers a high resistance to the passage of current (57). From the physical point of view, the impedance is a vector characterized by the R component on the axis of the abscissae and Xc on the axis of the ordinate. The angle created by the vector is defined as the phase angle (PA) and depends on the Xc (58).

### 2.4 Handgrip strength test

To assess muscle strength, we used the HGS test with a Jamar Plus dynamometer. The cutoffs of HGS are <30 kg for men and < 20 kg for women (59). The patient performed the test in a sitting position with a 90-degree flexed elbow. The researcher set up the instrument and asked the patient to squeeze as hard as possible for a few seconds. Three repetitions were conducted on the arm without the arteriovenous fistula, and the average value was considered for analysis.

## 3 Statistical analysis

Collected data were entered into an Excel spreadsheet (Microsoft, Redmond, WA, United States), and the analysis was performed using the Windows Social Science Statistics Package, version 25.0 (IBM_SPSS, Chicago, IL, United States). The descriptive statistics have been reported, after the confirmation of Kolmogorov–Smirnov test, according to the mean value ± standard deviation (for the parameters with normal distribution). For non-normal variables, we consider the median and the interval (minimum: maximum). In the first case, Student’s *t*-Testo (parametric test) was applied, while in the second case, the Wilcoxon test (non-parametric test) was applied.

Pearson’s correlation analysis was carried out for the evaluation of the possible linear relationship between the sarcopenia index (SI) through the calculation of the coefficient of Pearson and its statistical significance. To overcome the differences between the ages of the two groups, we preliminary applied a linear regression model (beta regression) to compare the estimators in terms of bias, variance, type-1 error, and power.

A *p* value of <0.05 was considered statistically significant.

## 4 Results

A total of 48 HD patients (34 men and 14 women) were recruited for this study and were divided according to the sarcopenia diagnosis (2). Epidemiological findings are shown in Table 1.

**Table 1:** Epidemiological findings of HD patients divided according to the sarcopenia diagnosis.

	Sarcopenia	No sarcopenia
Number of patients	36	12
Male/Female	25/11	9/3
Age, years	69.6 ± 1.6	56.3 ± 4.2
Dialytic vintage, months	36.5 (3–216)	42 (27–252)
Arterial hypertension, %	72.2	58.3
Diabetes mellitus, %	52.7	50

The laboratory results (Table 2) showed that patients without sarcopenia diagnosis had a transferrin value higher than those with sarcopenia diagnosis (*p* = 0.03). Furthermore, higher values were observed for total proteins (*p* = 0.04) and azotemia pre-dialysis (*p* = 0.05) in patients without sarcopenia diagnosis compared to those with sarcopenia diagnosis.

**Table 2:** Laboratory findings of HD patients divided according to the sarcopenia diagnosis.

Laboratory data	Sarcopenia	No Sarcopenia	*p* value
Albumin, gr/dL	3.9 ± 0.1	4.1 ± 0.1	ns
Lymphocyte, thousands/μL	1.2 ± 0.2	1.3 ± 0.2	ns
Transferrin, mg/dL	198.2 ± 12.5	249.0 ± 19.1	0.03
Total proteins, g/dL	6.4 ± 0.1	7.1 ± 0.2	0.04
Azotemia pre-dialysis, mg/dL	144.8 ± 5.7	169.5 ± 11.8	0.05
PLR	146.0 ± 16.9	158.3 ± 31.2	ns
NLR	4.6 ± 1.4	3.9 ± 0.7	ns
PTH, pg./mL	409.0 (153–1,253)	208.5 (100–317)	0.001
Vitamin D, ng/mL	23 ± 15,0	25 ± 17,1	ns
Phosphorus mg/dL	5,4 ± 1,6	5,2 ± 1,8	ns
Calcium mg/dL	8,7 ± 0,70	8,8 ± 0,6	ns

NLR, Neutrophil-to-lymphocyte ratio; ns, Not significant; PLR, Platelet-to-lymphocyte ratio; PTH, Parathyroid hormone.

We also analyzed the possible differences in the therapy of calcium–phosphorus metabolism (such as vitamin D analogs, phosphate chelating agents, and calcium mimetics) in sarcopenia and no-sarcopenia patients, highlighting no statistical differences between the two groups.

Anthropometric findings in the population of the study, divided according to the sarcopenia diagnosis, are reported in Table 3. Patients without sarcopenia showed higher values, in a statistically significant manner, of BMI (*p* = 0.01), PA (*p* = 0.02), FFM (*p* = 0.001), SI (*p* = 0.0001), SI adjusted for IMAT (*p* = 0.006), and femur *t*-score (*p* = 0.003) compared to sarcopenia patients.

**Table 3:** Anthropometric findings of HD patients divided according to the diagnosis of sarcopenia.

Anthropometric data	Sarcopenia	No Sarcopenia	*p* value
Body mass index, kg/m^2^	24 0.7 ± 0.6	28.2 ± 1.7	0.01
Hip/Waist	0.90 ± 0.01	1.0 ± 0.02	ns
PA, ° (BIA)	4.9 ± 0.2	6.0 ± 0.5	0.02
Fat mass, % (DXA)	22.6 ± 1.3	26.4 ± 3.7	ns
Fat mass, kg (DXA)	35.9 ± 1.5	33.9 ± 3.5	ns
Fat free mass, kg (DXA)	39.7 ± 1.2	49.2 ± 3.0	0.001
SI	5.9 ± 0.2	8.5 ± 0.9	0.0001
IMAT	0.90 ± 0.1	0.83 ± 0.3	ns
SI adjusted for IMAT	276.7 ± 43.0	576.4 ± 115	0.006
Handgrip strength test, kg	15.8 ± 1.5	22.0 ± 3.1	0.01
Column total *t*-score	−0.9 ± 0.2	−0.1 ± 0.3	ns
Femur *t*-score	−1.8 ± 0.2	−0.6 ± 0.5	0.003

BIA, bioelectrical impedance; DXA, dual-energy X-ray absorptiometry; IMAT, Intramuscular adipose tissue; PA, Phase angle; SI, Sarcopenia index.

**Figure 1:**
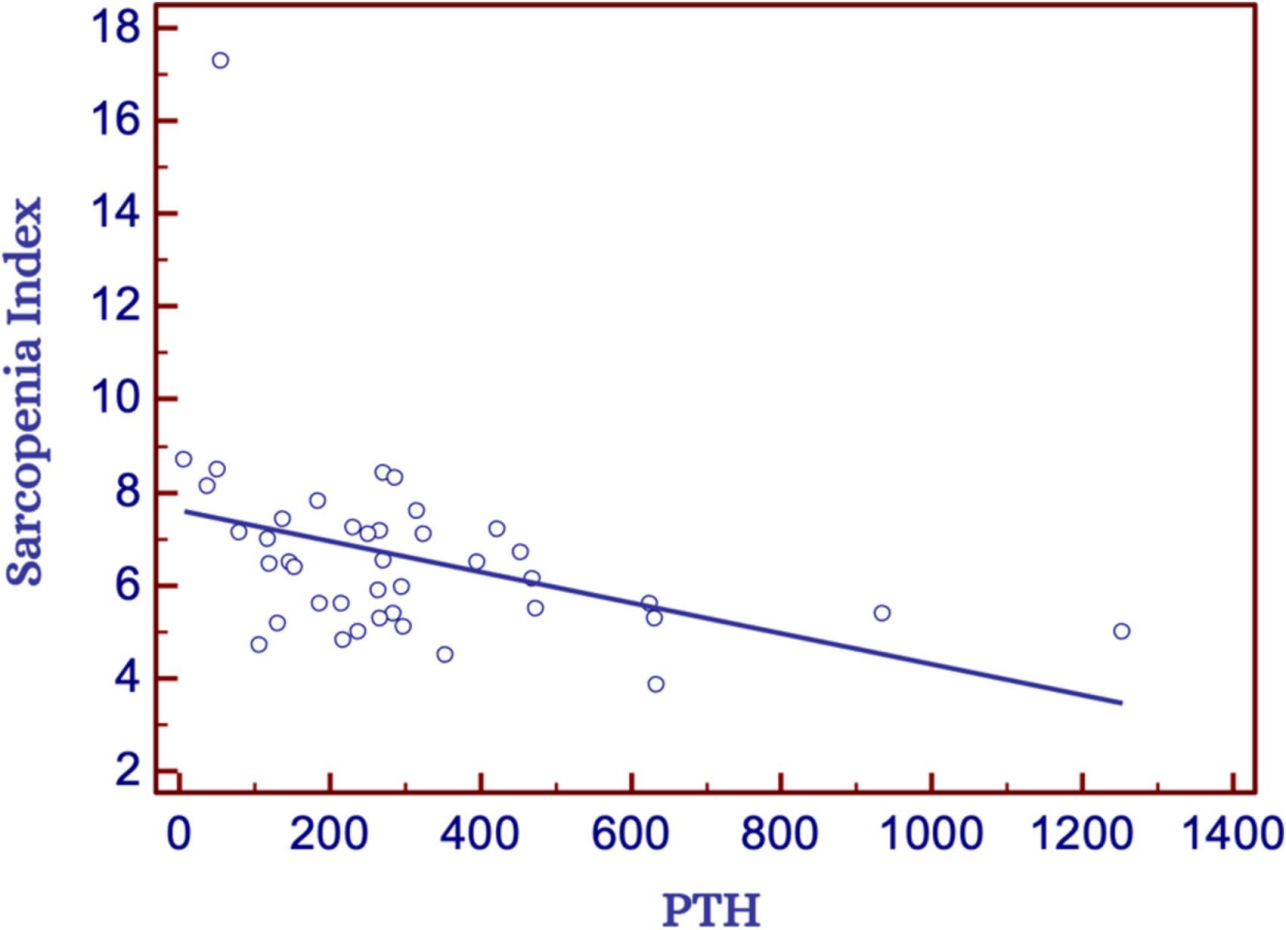
Correlation between sarcopenia index and PTH in the overall study population. PTH, Parathyroid hormone.

We also observed an interesting indirect correlation between the SI and PTH, as reported in Figure 1 (*p* = 0.00138 and *R*^2^ = 0.54).

**Figure 2:**
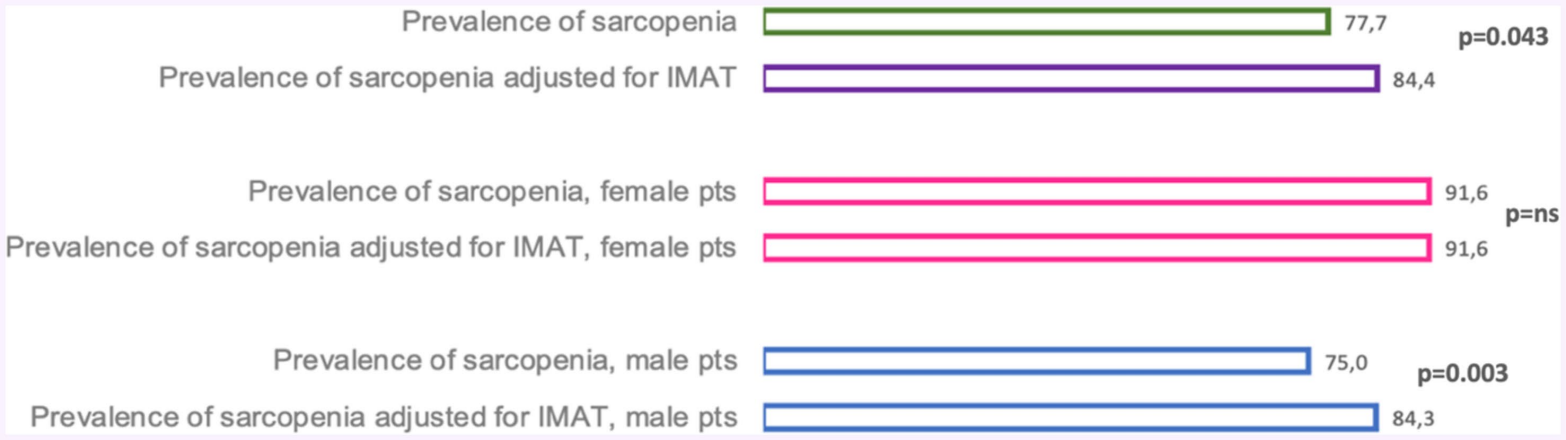
Sarcopenia index adjusted for IMAT in the overall study population and according to the male and female sex. IMAT, Intramuscular adipose tissue; ns, Not significant.

In Figure 2, we reported the prevalence of sarcopenia diagnosed according to SI compared to that obtained by SI adjusted for IMAT.

In the same figure, we showed, in both female and male patients, the prevalence of sarcopenia diagnosed by SI and the one obtained by SI, adjusted for IMAT. The difference in the prevalence of sarcopenia diagnosed using a standard index compared to that diagnosed using the SI adjusted for IMAT was statistically significant (*p* = 0.043).

For female patients, the prevalence of sarcopenia obtained using standard SI and the one detected using SI adjusted for IMAT was the same (*p* = ns). While, for male patients, when we adjusted the SI for IMAT, the prevalence was higher (*p* = 0.003).

## 5 Discussion

In our study, we enrolled a population of 48 HD patients and divided them into two subgroups based on the diagnosis of sarcopenia. In particular, 36 patients were affected by sarcopenia, namely 75% of our population. The mean age of sarcopenic patients was 69.6 ± 1.6 years vs. 56.3 ± 4.2 years for non-sarcopenic patients. The laboratory parameters highlighted a significant increase in transferrin, total protein, and azotemia pre-dialysis for non-sarcopenic patients. All these parameters are related to a better nutritional state. Several studies demonstrated that also serum transferrin concentration is a biomarker of nutritional state (60, 61).

Moreover, in our study population, we observed how sarcopenic patients had higher PTH values, demonstrating an inverse correlation between the SI and the serum PTH values. This correlation could be explained by the involvement of PTH in the browning of adipocytes through the activation of thermogenic genes (62). In fact, it is known in the literature that high levels of PTH can induce the browning of white adipose tissue, via the pathway involving the protein related to parathyroid hormone (PTHrP). This conversion leads to an increase in resting energy expenditure that, if not supported by an enhancement of caloric intake, induces a reduction of body weight, a loss of muscle mass, and, in the most severe case, cachexia (63, 64).

Furthermore, we highlighted a lower BMI in sarcopenic patients, according to previous literature studies conducted on different populations. In particular, Curtis et al. showed that in older adults, lower BMI values were significantly associated with a probable picture of sarcopenia, while higher BMI values were protective against sarcopenia (65). Another study, conducted on elderly women, demonstrated that higher BMI values were inversely correlated with low muscle mass (66).

In sarcopenic patients, the PA, obtained using the BIA, was lower than that obtained in non-sarcopenic patients. These data are supported by a recent study conducted by Wang et al. (67) in HD patients, which highlighted that PA should be useful in the detection of sarcopenia. The study was conducted on 241 HD patients, of which 28% were sarcopenic (diagnosis-based according to the Asian Sarcopenia Working Group). The sarcopenic patients showed lower PA values than the non-sarcopenic patients. This study concluded that PA should be helpful in predicting the risk of sarcopenia in HD patients. Moreover, as observed in our study population, these authors pointed out a lower HGS in patients affected by sarcopenia, compared to non-sarcopenic patients. This result is easily explained, as the reduction of muscle mass can induce a decrease in the HGS.

**Figure 3:**
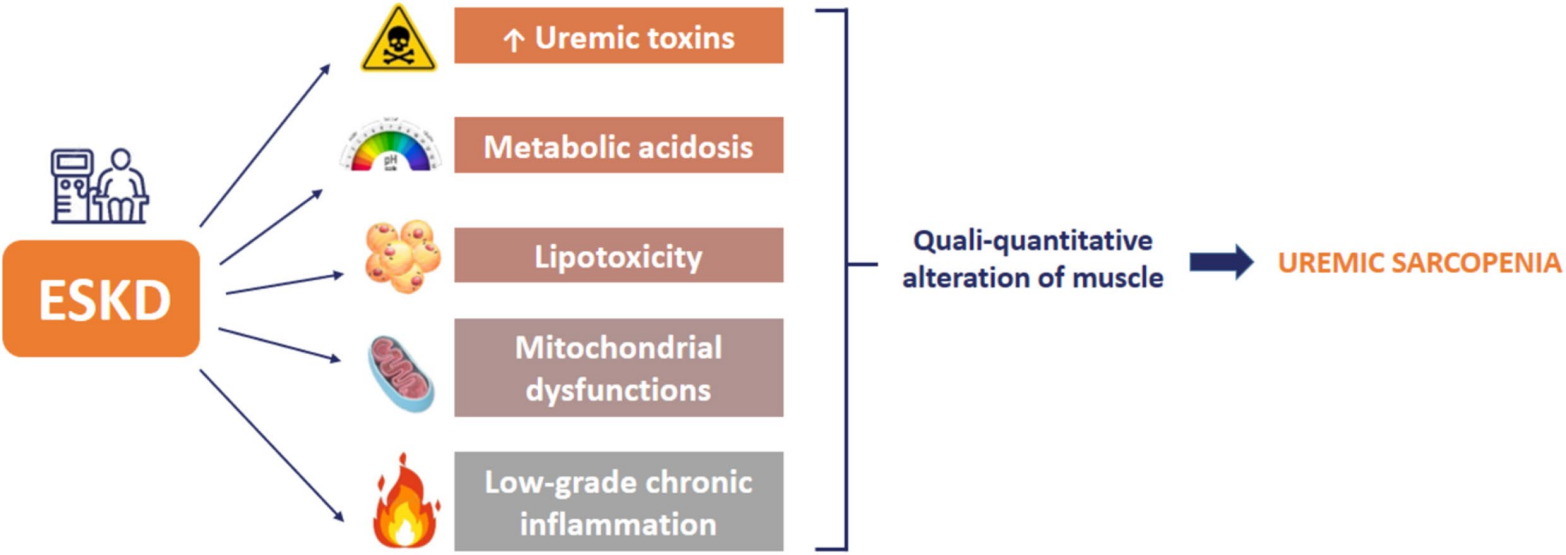
Sarcopenia risk factors in end-stage kidney disease (ESKD) patients.

In accordance with the literature (68), we observed a lower mean value of IMAT in non-sarcopenic patients than in sarcopenic patients, but the difference between the two groups was not statistically significant. This parameter is also related to the lower value obtained using the HGS test. In fact, the presence of adipose tissue in muscle induces a reduced contraction power of the muscle itself and, thus, a decreased functional capacity (69). In the general population, this phenomenon is mainly related to aging and physical inactivity. In CKD patients, this condition is ascribable to uremic risk factors such as low-grade chronic inflammatory status, metabolic acidosis, hyperparathyroidism, vitamin D deficiency, and mitochondrial dysfunctions (69, 70). The latter is caused by the accumulation of lipids and their derivatives either in and between muscle cells, inducing an impairment of fatty acid beta-oxidation and an enhancement of oxidative stress. These phenomena cause lipotoxicity that induces an increased production of pro-inflammatory cytokines, further worsening the systemic inflammation. This creates a vicious cycle, in which the inflammation induces muscle damage, which in turn exacerbates the inflammatory status (Figure 3).

In several chronic pathological conditions, it has been demonstrated that the loss of muscle strength and FFM are associated with lower bone mineral density (8, 71–73). In our population, we observed that sarcopenic patients had femur *t*-score values significantly lower than non-sarcopenic patients. In particular, sarcopenic patients present pathological femur *t*-scores, while non-sarcopenic patients present normal values. This observation is corroborated by a previous study conducted by Cheng et al. (74), in which the authors pointed out a possible link between “muscle-bone-lipid,” namely impairments of lipid metabolism and loss of muscle mass are associated with concomitant bone loss.

Moreover, it is important to underline the possible utility of SI adjusted for IMAT compared to the traditional SI. In fact, in our population, we highlighted a significant difference between the prevalence of sarcopenia diagnosed with the traditional SI compared to the SI adjusted for IMAT (*p* = 0.043).

Moreover, by dividing our population according to gender, we observed that these data are valid only for male patients. As reported in Figure 2 for female patients, we did not detect any statistical difference between sarcopenia diagnosed using the traditional SI and that diagnosed with the SI corrected for IMAT. We speculate that these data are related to the different adipose tissue expansions in male and female patients (75). In fact, the first ones are characterized by hypertrophic adipose tissue, while female subjects are characterized by hyperplastic adipose tissue. Moreover, we believe that it is necessary during diagnosis to define the sarcopenia category. In fact, sarcopenia can be categorized into: (i) pre-sarcopenia, as defined by the EWGSOP, i.e., by the presence of reduced muscle mass and normal muscle function; (ii) dynapenia, defined as normal muscle mass and reduced muscle function; (iii) and finally, full-blown sarcopenia. The latter has been defined by the diagnostic algorithm, drawn up by the Asian Working Group for Sarcopenia, as the simultaneous presence of low muscle mass associated with reduced muscle function (10, 76). The clinical management of chronic HD patients should therefore include the qualitative and quantitative evaluation of the muscle. In fact, the possible alterations present in the muscle should be studied in a specific and detailed manner, since the diagnosis of this comorbidity is fundamental for the prognosis and quality of life of ESKD patients.

The limitation of this study is the small sample size. In the future, it would be interesting to expand the population of enrolled patients to confirm the results obtained on a larger study population. The major strength is the originality of the study. In fact, for the first time in literature, the possible predictive value of IMAT on sarcopenia in an HD population has been examined, obtaining promising results.

## 6 Conclusion

In our study, we observed several interesting results, but the most important seems to be the indirect correlation between PTH and SI. In fact, these data corroborate new theories, already highlighted by other authors in the literature, in which PTH seems to play a central role in the cachexia genesis. Moreover, the SI adjusted for IMAT appears to be a reliable parameter, particularly for male patients, and is useful for the early identification of those at risk of developing US. This adjustment could facilitate the timely implementation of targeted therapeutic strategies.

## Data Availability

The raw data supporting the conclusions of this article will be made available by the authors, without undue reservation.
